# Supplementary material for: Prenatal and Postnatal Hair Steroid Levels Predict Post-Partum Depression 12 Weeks after Delivery
Source: J Clin Med. 2019 Aug 23;8(9):1290. doi: 10.3390/jcm8091290 (PMC6780455; doi:10.3390/jcm8091290)
Supplement: Supplementary file 1 [file jcm-08-01290-s001.pdf]

Table S1. Overview of the quality check, as described in the Systematic Assessment of Quality in Observational Research (SAQOR).

	Dimensions	Present Study	Scores	Sub/Total Score
	**Sample**			
1	The sample is representative of the population from which is was drawn	Yes; a total of 495 women 12 weeks after delivery were screened.	1	
2	The source of the sample is clearly stated	Yes; all participants gave birth at the Fatemieh Hospital (Hamadan) and attended the hospital for routine check	1	
3	The sampling method is described	Yes; participants were consecutively assessed and divided into participants with or without post-partum depression	1	
4	The sample size is appropriate to determine statistical significance for primary outcomes	Yes, a power calculation was performed to estimate the minimum sample size	1	
5	Entry criteria and exclusions are stated and justified	Yes, inclusion and exclusion criteria are clearly stated	1	
		Sample: total score:	5 out of 5	5/5
	Control/comparison group			
5	Control group is included	Yes, 50 women 12 weeks after delivery without post-partum depression	1	
6	The control group is easily identifiable	Yes, see text and Table 2 for description of the control group	1	
7	The source of the control is explained and appropriate	Yes; controls are recruited from the same hospital and assessed by the same medical and nursing staff	1	
8	Controls are matched or randomized	Controls are matched	1	
9	Statistical difference between cases and controls have been controlled for	Yes, see Table 2; women with or without post-partum depression do not statistically differ as regards age, gestational age, and neonates' Apgar score 10' after delivery	1	
		Control/comparison group total score:	5 out of 5	10/10
	Quality of exposure/outcome measurements			
10	Adequate assessment of exposure	N/A	0	
11	Adequate measure of outcomes	Yes, hair steroids	1	
		Quality of exposure/outcome measurements total score:	1 out of 2	11/12
	Distorting influences			
12	Key confounder 1 (MDD) is controlled for/taken into account in the design or analysis	Yes, though scores of MDD/PPD are also outcome variables	1	
13	Key confounder 2 are controlled for/taken into account in the design or analysis	Yes, preliminary calculations showed that age, gestational age and infants' Abgar index did not differ between the two groups	1	
14	Other potential confounders are controlled for/taken into account in the design or analysis	Yes, to avoid possible borderline self-ratings of post-partum depression, inclusion and exclusion criteria were defined in a restrictive fashion	1	
		Distorting influences total score	3 out of 3	14/15
	Reporting of data			
15	Explanation for missing data is given	Yes, though, no missing data did occur	1	
16	Data are clearly and accurately presented including CI were appropriate	Yes, all descriptive and inferential statistical indices are reported (see Table 3); effect sizes are reported for *t*- and *F*-test following Cohen [2]	1	
		Reporting of data total score	2 out of 2	16/17
	Overall conclusion of the quality			HIGH
